# A mixed method evaluation using the RE-AIM framework of a student-led community-based cardiovascular disease screening clinic in an urban community setting

**DOI:** 10.3389/fpubh.2026.1757096

**Published:** 2026-02-03

**Authors:** Angela Long, Matthew Cooper, Charlotte L. Richardson, Hamde Nazar

**Affiliations:** 1 School of Healthcare and Nursing Sciences, Northumbria University, Newcastle upon Tyne, United Kingdom; 2 National Institute for Health and Care Research Newcastle Patient Safety Research Collaboration, Newcastle University, Newcastle upon Tyne, United Kingdom; 3 School of Pharmacy, Newcastle University, Newcastle upon Tyne, United Kingdom

**Keywords:** cardiovascular screening, community clinic, public health evaluation, RE-AIM evaluation framework, student-led clinic

## Abstract

**Introduction:**

Evaluations of public health interventions often prioritise outcomes while neglecting contextual and implementation factors essential for sustainability. Using the RE-AIM framework (Reach, Effectiveness, Adoption, Implementation, and Maintenance), this study assessed the Young@Heart (Y@H) student-led cardiovascular disease (CVD) screening clinic—a community-based initiative that simultaneously delivers preventive health services and experiential learning for undergraduate pharmacy students.

**Methods:**

A concurrent mixed-methods case study was conducted across organisational, service, and individual levels over 12 months. Data sources included semistructured interviews with academic staff, patients, and external stakeholders; focus groups with student volunteers; service-activity data from 1,152 clinic attendees; and 20 fidelity assessments of service delivery. Quantitative and qualitative data were analysed independently, then integrated using the RE-AIM framework to triangulate findings across datasets.

**Results:**

Reach: the clinic attracted 1,152 participants (mean age 53 years), with representation from all socioeconomic deciles and 31% from the most deprived quintiles, demonstrating strong accessibility but limited engagement from younger adults. Effectiveness: High rates of modifiable risk were detected (44% elevated blood pressure, 62% overweight/obese, 36% cholesterol >5 mmol/L). Significant pre-post gains in self-reported motivation for dietary and physical-activity change (*p* < 0.001) aligned with qualitative reports of increased awareness and intention to act. Adoption: Stakeholders and participants valued the clinic’s dual educational–public health role; however, formal referral rates were low (9%), highlighting weak system integration. Implementation: fidelity checks showed >80% adherence in 18/20 observations, indicating strong interpersonal delivery but procedural inconsistency in referral and signposting practices. Maintenance: participants and stakeholders perceived the model as sustainable if embedded within curricula and supported by stable funding, though absence of follow-up data limited assessment of long-term behavioral maintenance.

**Conclusion:**

Applying the RE-AIM framework provided a comprehensive evaluation of the Y@H clinic, evidencing its accessibility, effectiveness, and educational value. While strong interpersonal delivery and measurable health impact were achieved, structural limitations in referral systems and follow-up impede sustained outcomes. Strengthening cross-sector referral pathways, standardising procedures, and embedding routine evaluation will be essential to ensure scalability, equity, and long-term sustainability of this innovative student-led community health model.

## Introduction

The evaluation of public health interventions typically focuses on the effectiveness of intended consequences, and fewer evaluations are focused on implementation, program reach (who is the intended audience), assessment of contextual factors affecting integration within wider organisation, community or systems, and fidelity of delivery. This can lead to the intervention’s mechanisms of change being assumed, and the unintended consequences missed, causing issues with replicability and future practice and wider-scale adoption (1).

The RE-AIM (Reach, Effectiveness, Adoption, Implementation and Maintenance) framework offers a robust evaluation model and encompasses five key dimensions for evaluating program impact at the setting and individual level (2).

- Participant engagement in terms of numbers and demographic representation (Reach),
- Impact on beneficial and negative outcomes including costs and quality of life (Effectiveness),
- Uptake by settings and providers (Adoption),
- Delivery fidelity and modifications (Implementation),
- Integration into routine practice (Maintenance) (3).

The framework can be used in the design, implementation and evaluation of interventions, allowing findings to be standardised across different contexts. This therefore allows for the reporting of factors influencing receptivity and sustainability (3). This provides a grounding in evidence-informed decision-making, and not assumptions about mechanisms of change or replicability.

The Young@Heart clinic, is a community-based student-led cardiovascular disease (CVD) screening clinic (4–6). This clinic is an innovative work-based learning opportunity for the training undergraduate pharmacy students, while achieving a parallel purpose of addressing health inequalities within the local community. This intervention has been evaluated for its feasibility, its impact on student learning, and its impact on patients. Finding demonstrate that the student-led clinic is feasible to deliver and engage with members of the public and has positive impacts on student learning and development and on the patients partaking in the screening intervention (4–6). Individually these studies provide findings that may appeal to different audiences and can be used as evidence of intervention effectiveness. However, these separate studies fail to inform future intervention designers and implementers about the collective impact of the intervention within the context it resides, the mechanisms of change, and challenges related to sustainability of Y@H as an intervention (1).

The aim of this study was to use the RE-AIM framework to comprehensively assess the student-led cardiovascular screening clinic for the purpose of effective dissemination and wider implementation.

## Methods

### Clinic overview

The clinic intervention has been outlined previously (4–6). For context, the Young@Heart clinic is a CVD screening intervention delivered by undergraduate pharmacy students under the supervision of academic pharmacists. Operating on a walk-in basis, the clinic is located in an indoor city centre market and offers measurements of height and weight (Body Mass Index, BMI), finger-prick cholesterol (total cholesterol), random blood glucose and blood pressure to members of the public to assess CVD risk. Clinic patients are offered lifestyle advice and referrals to relevant services (NHS or community based) where appropriate, e.g., smoking cessation support.

### Design

This was a single mixed-method case study adopting a concurrent triangulation design (7). Triangulation is the use of multiple approaches, data sources or methods to study a single phenomenon. The goal is to increase validity, credibility and depth of understanding of the findings. There are often multiple dimensions within an individual case study, so interrogation across a range of methods and integrating the findings will support the convergence to an interpretation of the phenomenon that is more holistic and less impacted by biases that are inherent with individual research methods (8, 9). This single case study design was used as no other student-led clinic of this nature is known to the research team. Also, the aim of undertaking this in-depth multi-perspective evaluation is to support the design, adoption and implementation of student-led clinic models at other sites and contexts.

Due to the complex nature of the intervention and its components, data were collected and analysed across three levels: the organisation, the service delivered, and the individual. At the organisational level, the units of analysis included the academic supervisors and stakeholders from onward referral services. At the service level, data was collected from clinic service activity. At the individual level, units of analyses included students delivering the service and patients receiving the service. Five sources of data were interrogated over a 12-month period which included: semi-structured interviews with academic staff, patients and external stakeholders; focus groups with students; activity data from the clinic; and fidelity checks of service delivery. Quantitative and qualitative data were collected concurrently and analysed independently with a short time frame to mitigate for any developments or changes in the case. Table 1 aims to demonstrate how the different data sources were rationalised against the domains of the RE-AIM framework.

**Table 1:** The research questions and sources of data that were interrogated across the domains of the RE-AIM evaluation.

Domain and research question	Detail	Source of data
Reach: how does the clinic engage members of the public in relation to the demographics of the population?	Demographics of patients *vs.* that of the local area	Clinic activity data
	Acceptability and motivation for clinic uptake	Patient surveys and interviews
Effectiveness: how effective is the clinic at achieving its public health aim?	Outcomes of check; clinical readings, *etc*. to identify risk	Clinic activity data
	Rates of behavioral intention change	Patient survey
	Student, patient, staff and stakeholder feedback	Interview data across students, patients, staff and stakeholders
Adoption: how does the clinic set up support intervention delivery? What are the barriers of adoption of the intervention?	Rates of referral to onward services	Clinic activity data
	Barriers and facilitators to adoption	
		Interview data across students, patients, staff and stakeholders
Implementation: how is the intervention implemented; what are the barriers and facilitators?	Fidelity of service delivery	Clinic activity data
	Fidelity of referral	
	Barriers and facilitators to implementation	Observational checklists
		Interview data across students, patients, staff and stakeholders
Maintenance: what are the long term effects of the intervention? What is required to support the longevity of the intervention?	Sustainability plan and organisational commitment	Interview data across students, patients, staff and stakeholders

Ethical approval was granted through institutional ethical procedures (Ref: 25658/2022).

### Qualitative data collection

Academic staff (*n* = 5) were emailed an invite to take part in the study with an accompanying participant information sheet and online consent form to complete. Interviews were undertaken in person or over Microsoft Teams at a mutually convenient time. Staff were asked about their thoughts and experiences of the clinic service model to explore barriers and facilitators to delivery and views on patient/student experience.

Patients accessing the clinic service were invited by the students at the end of the health check to participate in an interview. Those expressing interest were sent a participant information sheet and a consent form *via* email. Those completing the consent form were contacted to arrange a mutually agreeable time and mode of interview (telephone, virtual or in-person) to be conducted approximately two- and eight-weeks after their clinic consultations. The topic guide was informed by the Health Belief Model (10) to explore experiences and motivations of attending the clinic and any subsequent behavioral changes. The interviewer was independent from the clinic delivery staff to reduce social desirability bias.

Managers and key contacts within stakeholder organisations (referral partners) were invited *via* email to take part and share their views on the referral process. All interviews were carried out over Microsoft Teams at a mutually convenient time. The semi-structured guides were developed to offer insight into the maintenance of the health check programme and explore whether referrals from the health check clinic could become part of their organisational practice (3). Participants were asked questions regarding the services their organisation offers, their perceptions on the health check service and thoughts on the current and future referral pathways.

Pharmacy students were invited to participate in a focus group at the close of the clinic sessions. Consenting students were included in a focus group in person within the clinic space once the clinic was closed to members of the public and no academic staff were present. Students were asked to share their perceptions and experiences of delivering the health check service and about their developing skills and professional identity.

All interviews and focus groups were audio-recorded and transcribed verbatim. Transcripts were exported into NVivo 15 to facilitate analysis. Data from staff, students and patients have been reported individually and published elsewhere. Data saturation was achieved across all interviews and focus groups as described in those individually reported studies. In this prior work, qualitative data were subjected to thematic analysis (11) and the data from patients were analysed utilising thematic framework analysis, where the Health Belief Model was the guiding framework (10).

### Service activity and intention to change data

Data collected from the patient health check consultation was entered by students on to a Microsoft Form. Data included demographic information (gender, age, place of residence by postcode, self-reported ethnicity), health and lifestyle behaviors (e.g., dietary and exercise habits, smoking status and alcohol consumption), clinical measurements (BMI, blood pressure, total cholesterol, blood glucose). As part of the consultation, lifestyle advice was provided and information about any referrals or signposting conveyed. Patients were asked about their intention to make changes to lifestyle or health behaviors pre- and post-health check using a 7-point Likert scale [1—Extremely unlikely, (no intention to change) to 7—Extremely likely, (strong intention to change)]. Anonymised service activity data from the health checks were exported from Microsoft Forms into an Excel spreadsheet at the end of clinic provision (21st March 2025) and SPSS Statistics 29 for analysis. This data has been individually interrogated, reported and published elsewhere (5).

### Fidelity checks

To assess the consistency and quality of health checks delivery by students, fidelity assessments were conducted, evaluating how closely the delivery aligned with the clinic’s standard operating procedures (SOPs). One researcher conducted all fidelity checks so there were no need for inter-rater reliability and consistency checks. The four key areas of health check delivery included background and consent, social/life history, clinical measures, lifestyle advice/signposting (12) described adherence as a key measure of implementation fidelity, reflecting how closely an intervention is delivered as planned in terms of its content, frequency, duration, and coverage. Measuring adherence involved assessing how much of the intended content was delivered and how often. Components of the health check were scored either 0 (criteria was not introduced or discussed), 1 (Criterial was partially delivered) or 3 (criteria fully delivered). However, delivering every component may not be essential for success (13); an intervention can still be effective if its core, essential elements are delivered consistently. A threshold of >80% adherence to protocol was considered to demonstrate good fidelity (14).

### Integrated analysis using the RE-AIM framework

For this study, transcripts from students, staff, patients and external stakeholders were reanalysed using framework analysis (15) but, following familiarisation, initial codes were mapped against the RE-AIM criteria. Data were charted individually for each participant and interpretation followed within and between subgroups until a consensus was reached on final themes.

Interview data provided the richest information across the RE-AIM domains, so synthesis commenced through the development of initial themes. Qualitative data were triangulated within each subgroup population. The strength of convergence was judged on the frequency and extensiveness of overlapping themes. Subgroups were anticipated to have the strongest convergence due to their shared experience and understanding of the phenomenon. Triangulation across the subgroup data was repeated using these initial themes to identify differences across the case. Convergence across subgroups was identified when at least two of the three subgroup participants referred to a theme. Evidence of divergence were similarly expected across subgroups given their differing perspectives.

Evidence from the service activity and fidelity checks were then integrated into each RE-AIM dimension where applicable and triangulated with established themes. The extent of convergence and/or divergence between data sets was assessed and reported. Service activity data contributed to assessing Reach, Effectiveness and Adoption, and the fidelity check provided evidence for Effectiveness and Implementation.

## Results

### Qualitative data

All five staff members (three female, two male) took part in semi-structured interviews conducted between February and March 2025, with a mean age of 26.6 years (SD = 1.01). Their professional experience ranged from 2 to 5 years (mean 3.6, SD = 1.02). Two interviews were held face-to-face, and three were conducted *via* Microsoft Teams. Interview durations ranged from 24 to 49 min (mean 34.5, SD = 7.6).

Out of six stakeholder organisations, four took part in the study and were interviewed in March 2025. One was a mental health organisation supporting people in the community, one was a family recovery service for drugs and alcohol, one charity organisation offering a weight management programme, and one organisation offering volunteering opportunities. Participant (2 male, 2 female) roles within the organisations ranged from chief executive officer (*n* = 2), service manager (*n* = 1) and office manager (*n* = 1). Interviews lasted between 25.30 and 30.51 min (mean = 27.80, SD = 2.99).

A total of 22 pharmacy students (15 female, 7 male) participated in three in-person focus groups held between January and March 2025. One group included eight students and the remaining two comprised seven each. Participants had a mean age of 20.1 years (SD = 0.86). Discussions lasted 28–33 min (mean 31.4, SD = 1.9).

### Service activity data

Students delivered health checks to 1,152 patients. Descriptive statistics, correlation analyses, chi-square tests, and mixed-design ANOVAs were used across different data variables. Detailed analysis is published elsewhere.

### Fidelity checks

Twenty fidelity assessments were carried out during clinics between November 2024 and January 2025 across different days and times of clinic delivery and with different groups of students. Eighteen of the checklists demonstrated good fidelity reaching >80% adherence to protocol items. The items where adherence to protocol was weaker was the appropriate referral/signposting made for patients based on their clinic data.

### Integrated framework and quantitative analysis

A triangulated framework analysis combining qualitative findings from students, staff, stakeholders, and patients with quantitative service-activity data was undertaken to examine the Young@Heart clinic through the RE-AIM lens. Quantitative results both corroborated and refined qualitative insights, providing a comprehensive understanding of the clinic’s performance across each RE-AIM dimension (Table 2).

**Table 2:** Integrated RE-AIM analysis: qualitative and quantitative convergence.

Domain	Key integrated insights	Convergence strength
Reach	Accessible, visible, and appealing to older adults; quantitative data show 31% from most deprived areas, indicating socioeconomic reach but age skew.	★★
Effectiveness	High detection of modifiable risk (BP 44%, BMI 62%, Cholesterol 36%); significant post-check motivational gains align with reported behavioral changes and learning outcomes.	★★★
Adoption	Interpersonal enthusiasm (students, patients) strong; only 9% signposted and 6% smoking-support uptake confirm weak systemic adoption.	★
Implementation	Excellent interpersonal delivery; quantitative evidence (BMI declined by 30%, low referral throughput) supports procedural inconsistency and resource constraints.	★★★
Maintenance	Sustained intent and self-reported short-term changes; no objective follow-up data available. Continued institutional support and improved system-level coordination is required.	★

### Reach

Across data sources, the clinics were perceived as highly accessible and approachable. Qualitative accounts emphasised the clinic’s informal environment as an effective enabler of public engagement while noting that attendance skewed towards older, health-aware adults. These impressions were supported by service data: among 1,152 attendees, the mean age was 53 years (range 18–93), and over half fell within the NHS Health Check age bracket (40–74 years). While this confirmed the age bias, additional analyses revealed broad socioeconomic coverage, with 31% of attendees from the most deprived quintiles and representation across all deciles of the Index of Multiple Deprivation. This suggests the service successfully reached individuals in deprived urban areas, partially countering assumptions that the clinic appealed only to the already health-engaged. Attendance by both sexes and diverse ethnic groups (78% White British; 9% Asian) further reflected the city’s population profile.

Collectively, the quantitative and qualitative data demonstrate moderate-to-strong reach, characterised by accessibility and inclusivity but limited by opportunistic recruitment and underrepresentation of younger working adults. The convergence across datasets therefore strengthens the interpretation that location and informality facilitated engagement, even though demographic diversity remains a future priority.

### Effectiveness

Qualitative evidence consistently described the clinics as clinically, educationally, and psychologically effective. Students and staff observed tangible clinical benefits, while patients and stakeholders emphasised reassurance, awareness, and intentional behavioral change. These observations are substantiated by service-activity data showing substantial detection of modifiable risk: 44% of participants presented with elevated blood pressure, 62% were overweight or obese, and 36% had cholesterol above 5 mmol/L. Fidelity checks verified students followed protocol in offering and providing the physical assessments appropriately.

Pre–post self-report surveys recorded a statistically significant improvement in motivation and intention to modify diet and physical activity (*p* < 0.001), with particularly large gains among individuals with higher body mass index values. These quantitative results directly reinforce participants’ descriptions of the clinic as a “wake-up call” or “mini MOT.” Importantly, the consistency of motivational gains across deprivation levels suggests that the intervention’s health-promoting effects were not limited by socioeconomic status, aligning with stakeholder perspectives on its potential to address inequalities.

Overall, the combination of subjective and objective data demonstrates very strong convergence. The clinics were effective in improving awareness, identifying risk, stimulating health-related intentions, and providing authentic learning experiences for pharmacy students.

### Adoption

Adoption exhibited high interpersonal engagement but limited systemic integration across datasets. Students, staff, and patients expressed enthusiasm for the model, citing mutual benefit, professionalism, and satisfaction. Stakeholders also conveyed willingness to collaborate and viewed the service as a promising public health gateway. However, both academic staff and stakeholders raised concerns about inconsistent referral processes and the absence of feedback mechanisms—an issue confirmed quantitatively.

Service data indicated that only approx. 9% (*n* = 97) of attendees were formally signposted or referred to external services, and uptake of smoking cessation support among smokers was approx. 6% (*n* = 8). While lifestyle advice was provided widely (healthy eating *n* = 679; physical activity *n* = 603), few referrals were made to services addressing social isolation (*n* = 54) or alcohol use (*n* = 7). These figures corroborate qualitative accounts of limited partnership adoption despite strong local enthusiasm. Students reported a lack of confidence and sufficient knowledge to effectively refer and signpost consistently. Staff observed this student reticence and emphasised the need for greater awareness of referral services and referral mechanisms with digital solutions to support this in the clinic.

The integrated analysis therefore reveals a dual pattern: strong adoption at individual and interpersonal levels, yet systemic fragility in operational pathways and inter-organisational coordination. The quantitative evidence thus refines and strengthens the interpretation that institutional and referral infrastructure, rather than willingness, constrained full adoption.

### Implementation

Implementation quality was uniformly praised across qualitative groups for its interpersonal fidelity—students’ empathy, clarity, and professionalism—and criticised for inconsistent process delivery. The quantitative dataset provided direct evidence of this inconsistency. Approximately 30% of attendees declined BMI measurement, a pattern reflecting both patient discretions, the sensitivity of weight-related discussions noted by academic staff and students and observations during fidelity checks. Despite substantial detection of cardiovascular risk, referral throughput remained low, confirming that procedural gaps affected continuity of care. Fidelity checks also illustrated that students were not offering appropriate signposting/referral to patients based on their expressed need or identified risk through the physical assessments.

Significant negative correlations between deprivation and health indicators (BMI *r* = −0.23; glucose *r* = −0.12) showed that participants from more deprived areas exhibited higher risk, reinforcing stakeholder recommendations to strengthen cross-sector referral capacity. These findings demonstrate how process weaknesses can disproportionately affect those most in need.

By combining datasets, the analysis indicates very strong convergence: both qualitative and quantitative evidence identify implementation strengths in human delivery but systemic deficiencies in workflow standardisation, referral documentation, and resource provision (e.g., printed leaflets, Wi-Fi reliability, digital form usability). Quantitative data thus lend empirical weight to qualitative observations of procedural inconsistency.

### Maintenance

Maintenance emerged as the least evidenced RE-AIM domain and is supported by more perceptual rather than empirical findings. Follow-up interviews with patients indicated short-term maintenance of lifestyle changes and strong intent to re-attend a health check; students and staff also viewed the model as sustainable if embedded within curricula and adequately resourced. However, no longitudinal behavioral or clinical follow-up of outcomes was captured in the service dataset, limiting quantitative verification of sustained change.

Staff, students and stakeholders expressed optimism about the sustainability of the Young@Heart clinic, but with varying emphasis on what was required to sustain it.

Students viewed the clinic as an invaluable, ongoing learning opportunity and advocated for its annual continuation. Staff regarded sustainability as contingent on adequate resources, supervisor support, and curricular integration, while stakeholders linked longevity to stable funding and cross-sector coordination.

This absence of objective patient follow-up weakens convergence on maintenance outcomes. Nevertheless, the alignment of perceived feasibility across stakeholder groups—combined with repeated patient intentions to revisit the clinic—suggests potential for long-term maintenance if systematic tracking and institutional support, and improved coordination are introduced.

### Integrated interpretation

Taken together, the combined qualitative–quantitative analysis shows high overall alignment across RE-AIM domains. The strongest convergence occurs in Effectiveness and Implementation, where quantitative indicators (clinical risk detection, motivation scores, process completion rates) directly reinforce qualitative perceptions of impact and delivery quality. Reach and Adoption demonstrate partial convergence: quantitative data broaden the reach narrative by evidencing socioeconomic inclusion but also quantify the limited referral activity that qualitative participants described. Maintenance remains conceptually supported but empirically under-evidenced.

Overall, the triangulated findings confirm that the Young@Heart clinic as an intervention delivers measurable community health benefit, promotes professional learning, and is well received across stakeholder groups. However, operational inconsistencies and weak referral systems constrain its scalability and integration within existing public health infrastructures. Addressing these procedural limitations and implementing longitudinal outcome tracking will be essential for future expansion and sustainability.

In summary, integrating qualitative and quantitative findings provides a robust, multidimensional evaluation of the Young@Heart clinic. The intervention demonstrates high acceptability, effectiveness, and delivery quality across diverse perspectives, while quantitative metrics substantiate perceived impact. Nonetheless, system-level adoption and maintenance mechanisms remain the key development priorities to ensure equitable reach, closed-loop referral pathways, and enduring population health benefits.

### Implications for future delivery

1. Targeted reach—quantitative identification of higher risk among deprived groups supports strategic outreach (evening/weekend or workplace clinics) to diversify attendance.
2. Systematic referral infrastructure—the 9% referral rate confirms the need for a unified, digital–paper referral portal and feedback loop to partner agencies.
3. Accessible information—integrate printed leaflets with QR options to accommodate older patients and those with limited digital literacy.
4. Outcome tracking—introduce a 30–90-day follow-up contact to quantify maintenance of behavioral change and enable ongoing RE-AIM evaluation.

## Discussion

This mixed-methods evaluation, guided by the RE-AIM framework, provides a comprehensive assessment of the Young@Heart student-led health-check clinics. By integrating qualitative findings from students, staff, stakeholders, and patients with quantitative service-activity data, the study offers a multidimensional perspective on effectiveness, implementation, and sustainability. Across data sources, the clinics were found to be accessible, well received, and effective in identifying modifiable cardiovascular risk, while also serving as a valuable experiential learning platform for students. However, system-level adoption and referral mechanisms remained underdeveloped, and long-term behavioral maintenance could not be verified in the absence of follow-up data.

### Comparison with existing evidence

#### Reach

The observed attendance patterns largely mirror national findings from NHS Health Check evaluations, which report strong participation among middle-aged and older adults but lower engagement from younger and working-age groups (16–18). Nevertheless, service data revealed attendance from all deprivation deciles, with approximately one-third of participants originating from the most deprived quintiles—suggesting that the market-based, walk-in format effectively reduced some socioeconomic barriers. This contrasts with prior studies highlighting persistent inequalities in the uptake of preventive health checks within primary care settings (18, 19). The findings therefore indicate that while community-based delivery can improve reach, further targeted outreach is needed to enhance demographic diversity and engage underrepresented groups.

#### Effectiveness

Consistent with the wider literature on community-delivered cardiovascular screening (6–9), the Young@Heart clinic achieved high detection rates for previously unrecognised risk, with 44% (*n* = 501) of attendees presenting with elevated blood pressure and over 60% (*n* = 500) classified as overweight or obese. These results align with earlier evaluations showing that opportunistic community checks identify significant unmet need and can prompt positive behavioral intention (20, 21). Participants’ accounts of reassurance and increased motivation are also congruent with evidence that brief, feedback-driven interventions can foster short-term lifestyle modification (21, 22). In addition, 47% (*n* = 545) of attendees were outside the recommended age category to qualify for a nationally commissioned health check (age 40–74 years) (23), potentially capturing a wider demographic than traditional screening programmes and highlighting the complementary role such initiatives may play alongside commissioned services in addressing identification of modifiable risk factors in younger and older age groups.

Importantly, the educational benefit observed among student deliverers—improvements in communication, empathy, and confidence—echoes outcomes from comparable student-led and interprofessional public-health initiatives (24–26). Together, these findings position Young@Heart as a dual-impact intervention, achieving measurable preventive health gains while contributing meaningfully to professional development.

#### Adoption

The dual pattern of high interpersonal engagement but limited organisational adoption observed in this study reflects a recurrent challenge identified in prior evaluations of community screening (14–16, 27–29). Despite enthusiasm and satisfaction among participants, only 9% (*n* = 97) of attendees were formally referred onward, and uptake of smoking-cessation support was 6% (*n* = 8). Similar referral shortfalls have been reported elsewhere, attributed to inconsistent communication between providers and the absence of feedback loops (28, 30). The present findings therefore reinforce calls for streamlined, closed-loop referral pathways linking community-based programmes with local health and voluntary-sector partners (27, 31). Establishing such mechanisms is essential to maximise the downstream benefits of opportunistic screening.

#### Implementation

Implementation quality was characterised by strong interpersonal fidelity but procedural inconsistency, closely aligning with prior research on community pharmacy and outreach-based health checks (31–33). Quantitative process data—such as 30% of attendees declining BMI measurement—illustrate this variability and resonate with qualitative reports of discomfort around weight-related discussions.

Limited access to printed materials and reliance on QR codes, which presented a particular barrier for some older attendees attempting to access follow-up resources, mirrored previously reported accessibility issues in older populations (32). The observed association between greater deprivation and higher clinical risk also supports evidence that implementation gaps can exacerbate inequalities when referral systems are weakest where need is greatest (18). Collectively, these findings highlight the requirement for a standardised operating protocol, consistent supervision, and integrated referral documentation to ensure delivery fidelity and equitable access.

#### Maintenance

As in the wider preventive-screening literature, long-term behavioral maintenance remains poorly evidenced (23, 34). Qualitative interviews suggested sustained motivation and small lifestyle adjustments at 8 weeks, yet objective follow-up data were unavailable, limiting inferences regarding longer term behavioral change. This gap reflects a broader challenge in NHS Health Check evaluations, where maintenance outcomes are rarely tracked (23, 34). Incorporating structured follow-up (e.g., at 3–12 months) could strengthen future assessments and demonstrate whether short-term intention translates into sustained health behavior change.

#### Contribution to the evidence base

This evaluation contributes novel, practice-based insights to the literature on student-led health interventions. First, it is among the few studies to assess a student-delivered health-check service operating in an urban market environment, demonstrating feasibility and acceptability beyond traditional healthcare settings. Second, the integration of qualitative and quantitative evidence provides a holistic understanding of how interpersonal quality, system design, and clinical outcomes intersect. Third, the triangulated findings quantify known implementation challenges—particularly referral bottlenecks and inconsistent standard operating procedures—thereby offering empirical precision to previously anecdotal concerns. These insights can inform the scale-up of student-led and community-based preventive services both regionally and nationally.

#### Strengths and limitations

A major strength of this study is its mixed-methods design, which combines detailed qualitative insights with objective service-activity data within a RE-AIM framework. This approach enhances interpretive depth and enables assessment of both individual experience and system performance. Triangulation across participant groups further strengthens the credibility of findings. However, several limitations must be acknowledged. The case study design enables transferability but does not infer generalisability. The opportunistic sampling of clinic attendees limits generalisability beyond those present in the market environment. Also, those attendees who agreed to participate in follow-up interviews may have been the most health conscious and thereby skew findings. Quantitative data capture was constrained by reliance on self-report for behavioral intentions and the absence of longitudinal follow-up measures. Additionally, although the study engaged diverse stakeholder groups, the perspectives of non-attenders were not included, leaving potential barriers to engagement unexplored.

#### Implications for practice and research

The findings provide actionable insights for both service improvement and policy development. To enhance Reach, targeted promotion through workplaces, community networks, and evening or weekend sessions may engage younger and underrepresented populations. Improving Adoption and Implementation requires the establishment of a unified referral infrastructure, including an integrated digital–paper platform with feedback loops to referring providers. Hybrid communication methods—combining printed information with digital resources—should be employed to ensure inclusivity. For Maintenance, embedding routine follow-up (*via* SMS, email, or phone) at 3–12 months could provide vital evidence of sustained behavioral outcomes and reinforce participant accountability.

From an educational perspective, the study supports the continued integration of student-led public health initiatives into healthcare curricula as authentic, experiential learning opportunities that simultaneously deliver community benefit. For policymakers, the findings highlight the potential for student-supported outreach models to complement traditional NHS Health Checks, expanding capacity and improving access in urban populations. Future research should employ longitudinal, controlled designs to evaluate maintenance outcomes and the impact of enhanced referral systems on population health metrics.

## Conclusion

This RE-AIM evaluation demonstrates that the Young@Heart student-led clinics were accessible, effective, and highly valued by participants while providing substantial educational benefit. This offers evidence to support this model as an effective strategy for workforce training and development and potentially preventive service expansion. Quantitative data confirmed the clinics’ capacity to identify modifiable cardiovascular risk and promote health-related motivation. However, inconsistent referral mechanisms and limited follow-up represent key barriers to long-term impact. Strengthening system integration, standardising procedures, and embedding routine evaluation will be critical to ensuring the sustainability and scalability of this innovative community health model.

## Data Availability

The raw data supporting the conclusions of this article will be made available by the authors, without undue reservation.
